# Supplementary material for: Simple Method for Apples’ Bruise Area Prediction
Source: Materials (Basel). 2021 Dec 25;15(1):139. doi: 10.3390/ma15010139 (PMC8745963; doi:10.3390/ma15010139)
Supplement: Supplementary file 1 [file materials-15-00139-s001.zip › Figures S1-S3.pdf]

materials

MDPI

Supplementary Materials

Simple Method for Apples' Bruise Area Prediction

Monika Słupska *, Ewa Syguła, Piotr Komarnicki, Wiesław Szulczewski and Roman Stopa

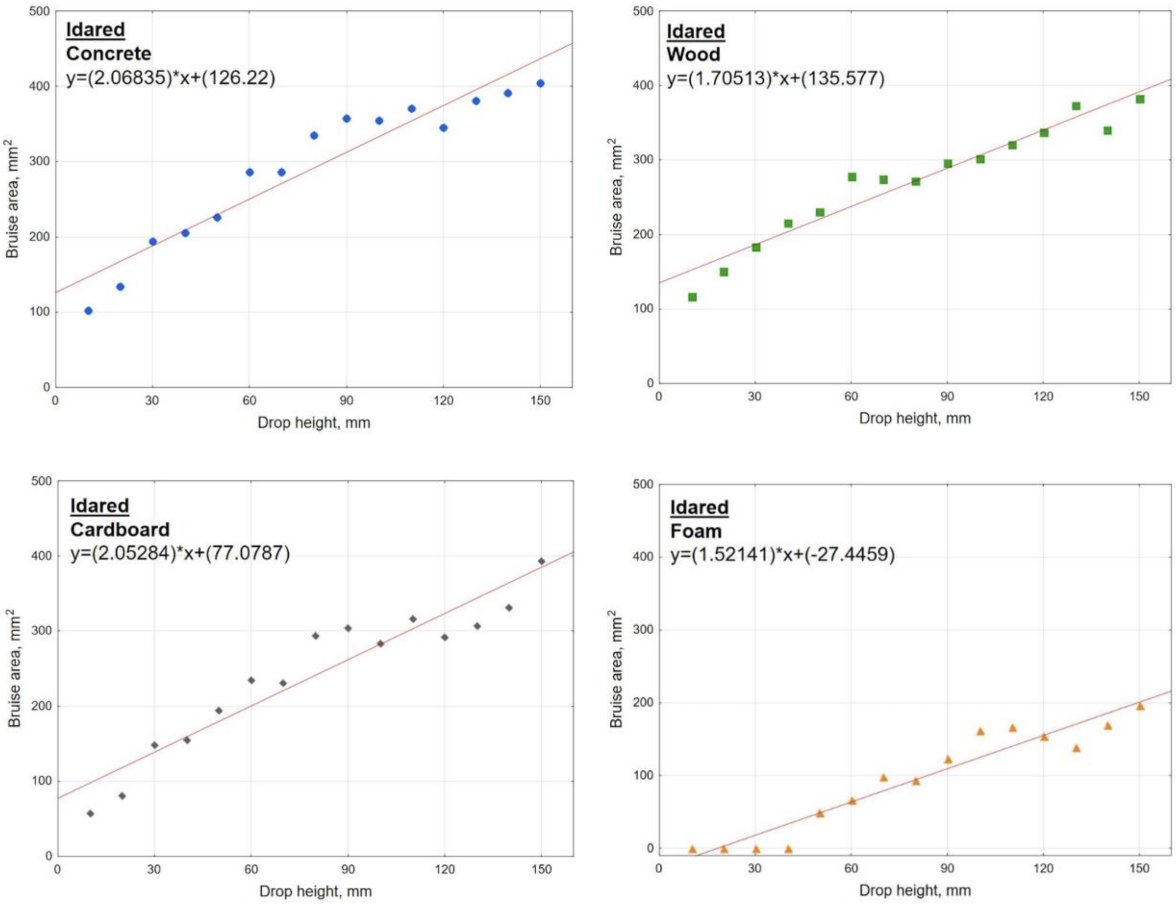

Figure S1. Dependence of bruised area of Idared apple as a function of drop height on different substrates.

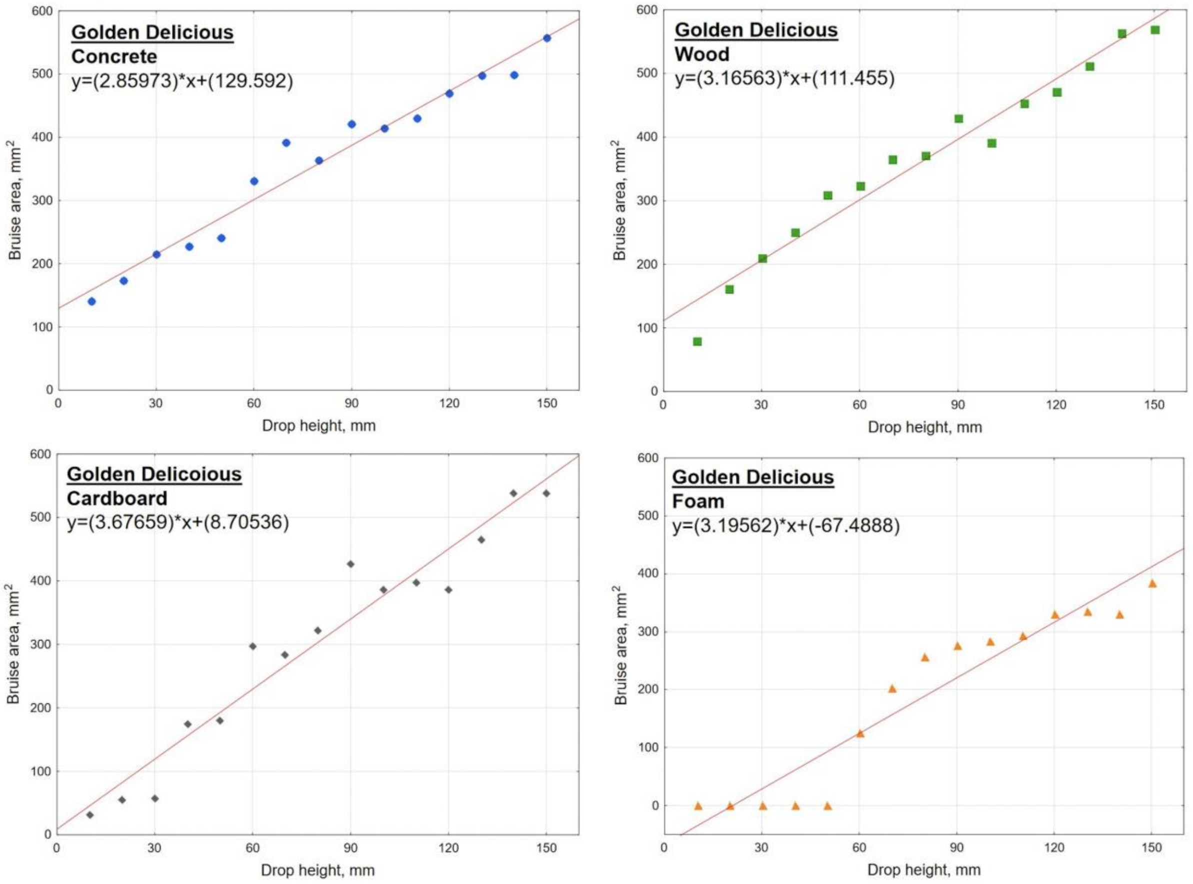

Figure S2. Dependence of bruised area of Golden Delicious apple as a function of drop height on different substrates.

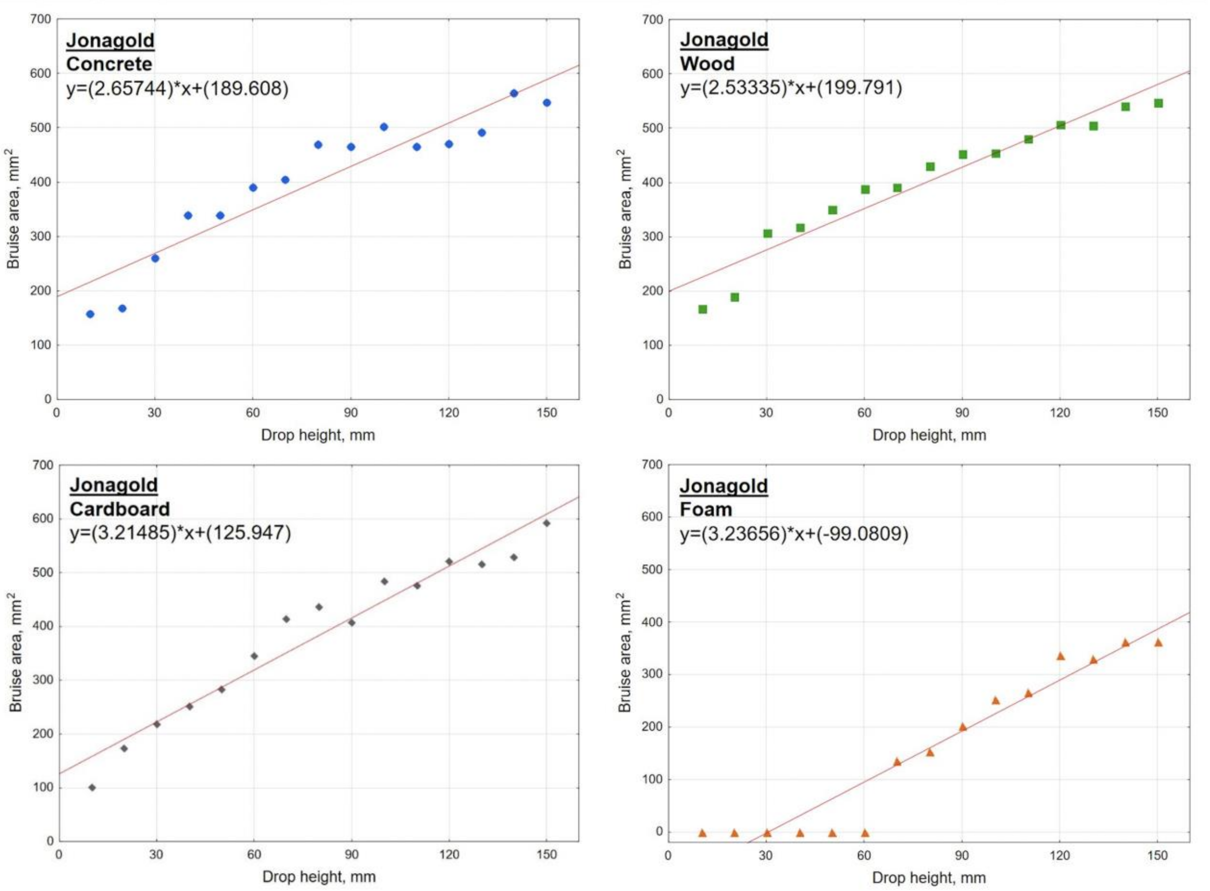

Figure S3. Dependence of bruised area of Jonagold apple as a function of drop height on different substrates.
